# Lombardy diagnostic and therapeutic network of thrombotic microangiopathy

**DOI:** 10.1186/s13023-022-02400-y

**Published:** 2022-06-23

**Authors:** I. Mancini, P. Agosti, M. Boscarino, B. Ferrari, A. Artoni, R. Palla, M. Spreafico, G. Crovetti, E. Volpato, S. Rossini, C. Novelli, S. Gattillo, L. Barcella, M. Salmoiraghi, A. Falanga, F. Peyvandi, Lombardy AREU TMA Network, Flora Peyvandi, Andrea Artoni, Barbara Ferrari, Roberta Palla, Ilaria Mancini, Pasquale Agosti, Marta Spreafico, Giovanni Crovetti, Elisabetta Volpato, Silvano Rossini, Anna Falanga, Laura Russo, Luca Barcella, Salvatore Gattillo, Chiara Novelli, Enrico Capuzzo, Marco D’Agostino, Porcari Moreno, Inzoli Alessandro, Pagani Ambrogio

**Affiliations:** 1 Department of Pathophysiology and Transplantation, Università Degli Studi Di Milano, and Fondazione Luigi Villa, Via Pace 9, 20122 Milan, Italy; 2 Fondazione IRCCS Ca’ Granda Ospedale Maggiore Policlinico, Angelo Bianchi Bonomi Hemophilia and Thrombosis Center, Milan, Italy; 3 Transfusion Medicine and Haematology Department, “A. Manzoni” Hospital, ASST-Lecco, Lecco, Italy; 4 SIMT, ASST Valle Olona, Busto Arsizio, Italy; 5 Division of Immunohaematology and Transfusion Medicine, ASST Grande Ospedale Metropolitano Niguarda, Milan, Italy; 6 Transfusion Center and Haematology Laboratory, Legnano Hospital, ASST Ovest Milanese, Legnano, Italy; 7 Immuno-Hematology and Transfusion Medicine Unit, San Raffaele Hospital, Milan, Italy; 8 Immunohematology Division, Hospital Papa Giovanni XXIII, Bergamo, Italy; 9 Unità Organizzativa Programmazione Della DG Welfare, Unità Organizzativa Programmazione Della DG Welfare Regione Lombardia, Regione Lombardia, Milan, Italy; 10 Department of Medicine and Surgery, University of Milan Bicocca, Milan, Italy

**Keywords:** ADAMTS13, Differential diagnosis, Incidence, Plasma-exchange, Thrombotic microangiopathy, Thrombotic thrombocytopenic purpura

## Abstract

**Background:**

Thrombotic thrombocytopenic purpura (TTP) is a rare, life-threatening thrombotic microangiopathy (TMA) requiring urgent treatment. Standardization of its diagnosis and optimal management is challenging. This study aimed to evaluate the role of centralized, rapid testing of ADAMTS13 in patients experiencing acute TMAs requiring plasma-exchange (PEX) and to estimate the incidence of TTP in a large Italian Region.

**Methods:**

We perfomed a cohort study in the frame of the project “Set-up of a Lombardy network for the study and treatment of patients undergoing apheresis”, including 11 transfusion centers in the Region. Consecutive patients referred from 2014 to 2016 with acute TMAs requiring PEX were enrolled. Centralized ADAMTS13 activity testing was performed at the Milan Hemophilia and Thrombosis Center within 24 h.

**Results:**

Forty-three TMA patients (44 events) were enrolled, of whom 35 (81%) had severe ADAMTS13 deficiency. Patients with severe ADAMTS13 deficiency were younger, mainly women, with a higher prevalence of autoimmune disorders and a lower prevalence of cancer. Clinical and laboratory characteristics of patients with and without severe ADAMTS13 deficiency largely overlapped, with a lower platelet count being the only baseline marker that significantly differed between the two patient groups (ADAMTS13 activity < 10% vs ≥ 10%: median difference of -27 × 10^9^/l, 95% CI − 37 to − 3). PEX treatment was initiated in all patients, but soon discontinued in cases without severe ADAMTS13 deficiency. In this group, the mortality rate was higher and no episode exacerbations or relapses within 6 months occured. The estimated average annual incidence of acute acquired TTP events was 1.17 [0.78–1.55] per million people.

**Conclusions:**

Severe ADAMTS13 deficiency distinguished two groups of patients with largely overlapping clinical features but different treatment and disease course. This study provides a feasible model implemented in a large Italian region for the practical clinical approach to TMAs and underlines the importance of urgent ADAMTS13 activity testing for an accurate differential diagnosis and therapeutic approach.

**Supplementary Information:**

The online version contains supplementary material available at 10.1186/s13023-022-02400-y.

## Background

Thrombotic thrombocytopenic purpura (TTP) is a rare, life-threatening thrombotic microangiopathy (TMA), with a reported annual incidence of 2 to 6 cases per million people [1–6] and a mortality of 10 to 20% even if properly treated [4, 7]. TTP is caused by the severe plasma deficiency of the von Willebrand factor (VWF) cleaving protease, i.e., the disintegrin and metalloproteinase with a thrombospondin type 1 motif member 13 ADAMTS13, caused in most cases by neutralizing autoantibodies (acquired immune-mediated TTP). As a result of ADAMTS13 deficiency, ultra-large VWF multimers accumulate in plasma and lead to multiple microvascular occlusions and organ tissue ischemia [8].

TTP belongs to a wide spectrum of disorders defined as thrombotic microangiopathies (TMAs), all characterized by thrombocytopenia, microangiopathic haemolytic anaemia and microvascular thrombosis in the terminal circulation with associated organ ischemia. In the early stages of acute TMA events, symptoms may be non-specific (e.g., weakness, confusion, headache, nausea). With the progression of the disease, as a result of the micro-thrombotic ischaemia, specific signs and symptoms of organ injury appear more frequently in the brain, heart or kidneys [4, 5, 7, 9–17]. However, several clinical and laboratory features may overlap, making differential diagnosis challenging [18]. Currently, ADAMTS13 activity testing in plasma is the key laboratory hallmark for the differential diagnosis of TMAs, because values below 10% of normal (i.e., ADAMTS13 severe deficiency) are diagnostic for TTP and support the initiation and continuation of therapy with plasma exchange (PEX) and immunosuppressors, nowadays with the adjunct use of the anti-VWF nanobody caplacizumab [19]. ADAMTS13 activity levels between 10 and 20% are considered borderline, alternative diagnoses should be seeked and treatment should be based on clinical judgement. Finally, ADAMTS13 activity levels above 20% of normal exclude a diagnosis of TTP, pointing towards other diagnoses and other therapies [19]. Moderately reduced to normal levels of ADAMTS13 activity are found in patients with other TMAs such as the complement-mediated hemolytic uremic syndrome, that benefits from eculizumab treatment. In non-TTP TMA patients, the measurement of ADAMTS13 would allow not only to consider a different diagnosis and treatment, but also to interrupt PEX when started, considering that this therapy is not free from adverse effects, some of which can be life threatening. In particular, plasma-related (anaphylaxis, anaphylactoid reactions, hypocalcemia/hypomagnesemia) and catheter-related events (infections, bleeding) have been reported [20].

A standardized diagnostic process and management of TTP/TMA patients is hampered by the rarity of the disease and the long turn-around time of ADAMTS13 testing, which might take days due to the technical characteristics of currently available assays, that are unsuitable for emergency laboratories and usually limited to specialized centers [21]. To promote the optimal management of TTP/TMA patients, the Italian Region of Lombardy set-up a network of transfusion and medical centers for the diagnosis and treatment of patients undergoing PEX. Among the study aims there was the evaluation of the clinical usefulness of centralized ADAMTS13 activity testing in patients experiencing acute TMA episodes requiring PEX. Accordingly, we report herewith the results of a comparative analysis of clinical and laboratory features, treatment and outcomes in TMA patients requiring PEX with and without severe ADAMTS13 deficiency. With this study we also aim at estimating the incidence of TTP in Lombardy.

## Methods

### Study design, patients and recorded variables

Between 2014 and 2016, we performed a cohort study in the frame of the project “Set-up of a Lombardy network for the study and treatment of patients undergoing apheresis” (“Costituzione di una rete lombarda per lo studio e il trattamento dei pazienti sottoposti a procedure di aferesi terapeutica”, Decreto n. 9269 di Regione Lombardia, 18/10/2012). The general project aimed at creating a regional network of tranfusional centers to standardize the protocols related to therapeutic apheresis and for assessing the feasibility and clinical value of centralized, rapid testing of ADAMTS13 activity in patients with a suspected diagnosis of TTP, the latter being the aim of the study herein reported. According to a survey conducted in 2013 in Lombardy hospitals, 34 out of 44 centers performed therapeutic plasma-exchange. Among these, 11 (32%) participated to the study herein reported. Based on the surveyed number of overall therapeutic plasma-exchange procedures performed in the previous year, these 11 centers were representative of 50% of all plasma-exchange procedures performed in Lombardy Region.

Consecutive patients presenting with acute TMA requiring PEX enrolled at 11 transfusion centers in the Region between 2014 and 2016 were included in this study. The list of enrolling centers is included in the Additional file 1: Table S1. TMAs requiring PEX [22] were defined based on the concomitance of consumption thrombocytopenia, microangiopathic hemolytic anemia and the occurrence of microthrombosis-related ischemic symptoms. Peripheral blood samples were collected at presentation of the acute episode before any transfusional treatment and shipped to the coordinating center (Fondazione IRCCS Ca’ Granda Ospedale Maggiore Policlinico, Milan, Italy) for ADAMTS13 testing. The acute phase results of ADAMTS13 activity and anti-ADAMTS13 antibodies were returned to the participating centers within 24 h after sample reception.

Age, sex, body mass index (BMI), clinical history, disease triggers, clinical manifestations, main laboratory parameters, treatments and outcomes of acute events were recorded. Data on concomitant cancer or autoimmune diseases were also collected. Potential disease triggers (i.e., events occurring, such as infections or surgery, or drugs taken in the three months prior to the acute episode), were also recorded. Furthermore, we collected data on bleeding, cardiovascular, neurological, renal and systemic symptoms/signs as observed at the time of onset of the acute episode. Laboratory tests, performed during the acute phase before plasma therapy, included platelet count, hemoglobin, leukocytes, serum lactate dehydrogenase (LDH), total and indirect bilirubin, transaminases, creatinine and plasma fibrinogen according to Clauss method. Estimated glomerular filtration rate (eGFR) was calculated by means of the Cockcroft-Gault equation.

Treatments such as PEX, plasma infusion, immunoglobulins, corticosteroids, rituximab, antiplatelets and anticoagulants were also recorded. The short- and long-term outcomes of the acute event were death, clinical response (i.e., sustained normalization of platelet count and LDH after stopping PEX treatment), exacerbation (i.e., recurrence of thrombocytopenia and increased LDH occurring within 30 days after a first clinical response and requiring to restart PEX therapy), episode length (defined as the number of daily PEX procedures required to achieve the first clinical response) and relapse (i.e., a de novo acute TMA event occurring more than 30 days after clinical response in the context of previous acute event). Written informed consent was obtained from all subjects in accordance with the Helsinki Declaration.

### Sample collection and ADAMTS13 testing

Peripheral blood samples were collected in vacutainer tubes containing 3.2% sodium citrate as anticoagulant. Citrated plasma samples were obtained by centrifugation for 20 min at 3200 g and 4° kept frozen at -80 °C until shipment. ADAMTS13 testing included ADAMTS13 activity, ADAMTS13 antigen and anti-ADAMTS13 antibodies, which were measured using ELISA-based commercial kits from Technoclone (Vienna, Austria): the Technozym® ADAMTS-13 Activity assay for ADAMTS13 activity, the Technozym® ADAMTS-13 Antigen for ADAMTS13 antigen and the Technozym® ADAMTS-13 Inhibitor for anti-ADAMTS13 IgG.

### Statistical analysis

Age, sex, BMI, clinical history, disease triggers, clinical manifestations, laboratory parameters, treatments and outcomes were compared in the frame of the acute TMA episodes in patients with and without severe deficiency of ADAMTS13 activity (< 10% or ≥ 10%). Categorical variables were expressed as counts and percentages, continuous variables as medians and interquartile ranges (IQR). Differences in proportions and medians were evaluated with the chi-square or Fisher’s Exact tests (where appropriate) and the Mann–Whitney test, respectively. In order to estimate the annual incidence rate of TTP and of overall acute TTP events for each follow-up year, the number of incident TTP events or of all acute TTP events were divided by the number of people living in Lombardy (in terms of persons-years). Then the incidence was expressed as cases per million people with 95% confidence intervals (CI) and a mean value calculated for the three years of the study. To account for the fact that not all Lombardy transfusion centers participated to the study, we provided two additional estimates for the incidence of acute TTP events under the following assumptions: (i) being the 32% of hospitals with transfusion medicine services in the Region, the 11 recruiting centers are representative of one-third of the Lombardy population at risk, (ii) accounting for 50% of overall plasma-exchange procedures performed in the Region, the 11 recruiting centers are representative of half of the Lombardy population at risk. Statistical analyses were performed using R version 3.5.3.

## Results

Between 2014 and 2016, 43 patients for a total of 44 acute TMA events were evaluated. Thirty-five patients (81%) had at presentation a severe deficiency of ADAMTS13 activity (< 10% of normal value), 8 (19%) had values ≥ 10%. Overall, 30 of 44 were first acute TMA events (68%), 22 (63%) among patients with severe ADAMTS13 deficiency and 8 (100%) among those without.

Compared with patients with ADAMTS13 ≥ 10%, those with severe ADAMTS13 deficiency were younger, with a higher proportion of women, autoimmune disorders and a lower proportion of cancer (Table 1). Autoimmune thyroiditis and connective tissue diseases were the most frequent autoimmune disorders, whereas cancers were mostly hematological.

**Table 1:** Baseline characteristics of patients with and without severe ADAMTS13 deficiency

	ADAMTS13 < 10% (n = 35)	ADAMTS13 ≥ 10% (n = 8)	Difference of proportions/ median difference (95% CI)
Female sex, n (%)	23 (66)	3 (38)	28 (− 17–73)
Age at 1^st^ episode, median (IQR)	48 (29–60)	58 (50–79)	− 18 (− 31 to − 1)
Age at enrollment, median (IQR)	50 (40–63)	58 (50–79)	− 13 (− 28–3)
BMI, kg/m^2^, median (IQR)	25 (22–28)^a^	25 (23–26)	0.87 (− 2.56–4.57)
Autoimmune disease, n (%)	11 (31)	1 (13)	18 (− 16–54)
Cancer, n (%)	0 (0)	4 (50)	− 50 (− 92 to − 8)

^a^Available for 29 patients

*IQR* Interquartile range, *CI* Confidence interval

Ten out of 36 episodes with severe ADAMTS13 deficiency (28%) and 4 out of 8 episodes with ADAMTS13 ≥ 10% (50%) were triggered by infections (mostly oropharyngeal or gastrointestinal), whereas one episode of both groups was triggered by surgery.

Table 2 summarizes the main clinical and laboratory characteristics of patients with acute TMA events at the time of presentation, which showed a large overlap between the two groups of patients. Among all recorded parameters, platelet count was the only baseline marker that significantly differed between the two study groups: TMA patients with severe ADAMTS13 deficiency had lower platelet counts (median difference − 27 × 10^9^/l, 95% CI − 37 to − 3), most of them presenting with severe thrombocytopenia (i.e., < 30 × 10^9^/l). Patients with severe ADAMTS13 deficiency also showed a trend for a higher proportion of bleeding (42% vs 13%) and cardiovascular signs and symptoms (11% vs 0%) and a lower proportion of renal failure (11% vs 38%), compared with those with ≥ 10%.

**Table 2:** Clinical and laboratory characteristics events at of acute TMA presentation in patients with and without ADAMTS13 deficiency

	ADAMTS13 < 10% (n = 36)	ADAMTS13 ≥ 10% (n = 8)	Difference of proportions/ median difference (95%CI), *p*-value
*Clinical signs and symptoms*			
Systemic, n (%)	32 (89)	7 (87)	2 (− 25–28), 1
Hemorrhagic, n (%)	15 (42)	1 (13)	29 (− 6–65), 0.25
Neurological, n (%)	14 (39)	4 (50)	− 11 (− 57–35), 0.97
Renal, n (%)	4 (11)	3 (38)	− 27 (− 70–16), 0.19
Cardiovascular, n (%)	4 (11)	0 (0)	11 (− 7–29), 0.76
*Laboratory assays*			
Platelet count, 10^9^/l, median (IQR)	14 (10–25)	43 (34–51)	− 27 (− 37–3), 0.02
Platelet count < 30 × 10^9^/l, n (%)	31 (86)	2 (25)	61 (21–100), 0.01
Hemoglobin, g/dl, median (IQR)	8.5 (7.2–11.2)	7.6 (6.9–9.1)	0.9 (− 1.0–2.9), 0.35
LDH, IU/l, median (IQR) ^a^	1039 (832–1761)	1197 (836–1956)	− 113 (− 927–450), 0.67
Indirect bilirubin, mg/dl, median (IQR) ^b^	1.8 (1.2–2.9)	1.8(1.2–2.3)	0.2 (− 1.1–1.9), 0.79
Creatinine, mg/dl, median (IQR) ^c^	1.0 (0.8–1.3)	1.4 (0.8–2.0)	− 0.3 (− 0.9–0.3), 0.38
Creatinine < 2.26 mg/dl, n (%)	30 (83)	7 (88)	− 5 (− 34–26), 1.00
eGFR, ml/min, median (IQR) ^d^	79 (53–107)	50 (30–107)	17 (− 28–55), 0.38

Systemic symptoms/signs: fatigue, fever, headache, jaundice (defined as total bilirubin levels ≥ 2.5 mg/dl); neurological symptoms/signs: ischemic stroke, transient ischemic attack, seizures, cognitive status alterations, personality disorders, focal neurological signs; hemorrhagic symptoms/signs: skin bleeding (purpura, ecchymosis), mucosal bleeds (including epistaxis, hematuria, meno-metrorrhagia, gastrointestinal bleeding); cardiovascular symptoms/signs: acute coronary syndrome or myocardial infarction; renal symptoms/signs: acute renal failure

^a^Available for 35 events with ADAMTS13 < 10% and 8 events with ADAMTS13 ≥ 10%

^b^Available for 26 events with ADAMTS13 < 10% and 4 events with ADAMTS13 ≥ 10%

^c^Available for 33 events with ADAMTS13 < 10% and 8 events with ADAMTS13 ≥ 10%

^d^Available for 28 events with ADAMTS13 < 10% and 8 events with ADAMTS13 ≥ 10%

With regard to ADAMTS13 testing results, 94% of acute TMA events with severe ADAMTS13 deficiency had undetectable ADAMTS13 activity levels, a reduced ADAMTS13 antigen (< 0.6 ug/ml) and positive anti-ADAMTS13 antibodies (> 15 U/ml). Among patients without severe deficiency, 2 showed reduced ADAMTS13 activity levels (19% and 33%) and 6 normal activity levels (range 45–80%). Reduced ADAMTS13 antigen levels and positive anti-ADAMTS13 antibodies were found in 38% and 25% patients, respectively (Table 3).

**Table 3:** Results of ADAMTS13 antigen and anti-ADAMTS13 antibodies in patients with and without severely deficient ADAMTS13 activity

	ADAMTS13 < 10% (n = 36)	ADAMTS13 ≥ 10% (n = 8)
*ADAMTS13 antigen*		
Reduced, n (%)	34 (94)	3 (38)
Normal, n (%)	2 (6)	5 (62)
Titer, ug/ml, median (IQR)	0.1 (0.04–0.16)	0.66 (0.53–0.72)
*Anti-ADAMTS13 antibodies*		
Negative or borderline, n (%)	2 (6)	6 (75)
Positive, n (%)	34 (94)	2 (25)
Titer, U/ml, median (IQR)	63 (39–93)	18; 21^a^

^a^Individual data

Treatments and outcomes of acute TMA events are reported in Table 4. PEX treatment was initiated in all TMA patients but the treatment period was longer in patients with severe ADAMTS13 deficiency. Corticosteroids use was similar in the two groups, but rituximab was employed only in patients with severe deficiency. In particular, among the 14 episodes treated with rituximab, 6/14 (43%) were first events, of whom 4 exacerbated. Pertaining to outcomes, 3 TMA patients died (3% of patients with severe ADAMTS13 deficiency vs 25% without), 14 patients exacerbated and 6 relapsed at 6 months after the acute event, all from the patient group with ADAMTS13 activity below 10% (Table 4).

**Table 4:** Treatment and short- and long-term outcomes of acute TMA events in patients with and without ADAMTS13 deficiency

	ADAMTS13 < 10% (n = 36)	ADAMTS13 ≥ 10% (n = 8)	Difference of proportions/ Median difference (95% CI), *p*-value
*Treatment*			
PEX, median (IQR)	15 (7–20)	5 (4–7)	9 (2–14), 0.003
Corticosteroids, n (%)	32 (88)	6 (75)	13 (− 25–53), 0.64
Rituximab, n (%)	14 (39)	0 (0)	39 (15–62), 0.08
*Outcome*			
Median time to remission, median (IQR) ^a^	19 (7–24)	Na	
Death, n (%)	1 (3)	2 (25)	− 22 (− 60–16), 0.14
Exacerbation, n (%)	14 (39)	0 (0)	39 (15–62), 0.08
Relapse at 6 months, n (%)	6 (17)	0 (0)	17 (− 3–36), 0.50

^a^The difference between date of episode onset and the date on which PEX treatment was stopped (in days)

Based on the detection of severe ADAMTS13 deficiency, 35 patients (81%) were diagnosed with acquired TTP, whereas the remaining 8 patients were ultimately diagnosed with complement-mediated HUS (n = 3), TMA secondary to cancer (n = 3) or TMA of unknown etiology (n = 2). Therefore, we estimated an average annual incidence of acquired TTP (i.e., incident cases) and of all acute acquired TTP events in Lombardy region of 0.73 (95% CI 0.43 to 1.04) and 1.17 (95% CI 0.78 to 1.55) per million people, respectively (Additional file 1: Table S2). By assuming that the 11 recruiting centers were representative of one third or half of the Lombardy population at risk, the aforementioned figures increased to an annual incidence of 2.34 and 3.51 acute TTP events, respectively.

## Discussion

We compared the disease-related features of 43 consecutive patients admitted to 11 hospitals in the Italian region of Lombardy for an acute TMA episode requiring PEX therapy and stratified according to the plasma level of ADAMTS13 activity. Severe deficiency distinguished two groups of patients with largely overlapping clinical and laboratory characteristics but very different treatment and disease course, confirming the importance of ADAMTS13 testing for an accurate differential diagnosis and management of TMAs.

As previously reported in the literature, patients with severe ADAMTS13 deficiency were more likely to be young, female, with a history of autoimmune diseases but no previous cancer as compared with those with ADAMTS13 ≥ 10% [7, 23, 24]. At hospital admission, the clinical and laboratory characteristics of patients largely overlapped, with clinical manifestations better differentiating patients with ADAMTS13 activity below and above 10% being hemorrhagic symptoms and renal failure. Neurological involvement, which is frequently associated with TTP, was not different between the two groups. These findings confirm previous data from European and US TMA registries, which reported a 44% lower proportion of acute renal failure in patients with severe ADAMTS13 deficiency [7] and a similar prevalence of 40–60% of neurological manifestations regardless of the ADAMTS13 activity level [7, 23, 24].

Regarding the laboratory parameters at the time of presentation of the acute TMA event, only platelet counts were significantly different between the two patient groups, a severe thrombocytopenia (count < 30 × 10^9^/l) being present in almost all patients with severe ADAMTS13 deficiency and in only 25% of those with ADAMTS13 ≥ 10%. More severe thrombocytopenia is likely to explain the higher prevalence of bleeding observed in patients with severe ADAMTS13 activity deficiency.

A markedly reduced platelet count is a cornerstone of the diagnostic scores employed to predict severe plasma deficiency of ADAMTS13 and thus the likelihood of TTP diagnosis [23, 25]. Another component of the scores is serum creatinine, with values lower than 2.26 mg/dl or 2 mg/dl predicting severe ADAMTS13 deficiency in the French or PLASMIC scores. Severe thrombocytopenia was clearly associated with severe ADAMTS13 deficiency also in the present study, but this association was not found for creatinine, perhaps owing to the small sample size, especially for patients with ADAMTS13 ≥ 10%. Unfortunately, we could not calculate the PLASMIC score, which can be used to select patients that would benefit from plasma-exchange. However, a recent meta-analysis by Paydary and colleagues demonstrated that the PLASMIC score can support differential diagnosis by excluding TTP but, due to low specificity and positive predictive value, is insufficient to confirm TTP diagnosis [26]. Hence, ADAMTS13 activity measurement remains necessary.

A criterion for inclusion in this study was the indication of PEX therapy [22]. Hence, the enrolled patients were all likely to be characterized by a high pre-ADAMTS13 testing probability of TTP, that perhaps explains the disproportion between the two patient groups (80% of patients had severe ADAMTS13 deficiency). Notwithstanding these issues, our findings underline the high degree of overlapping features in patients with a clinical suspicion of TTP and the importance of rapid measurement of ADAMTS13 activity for an accurate differential diagnosis and optimal management.

Both laboratory markers, antigen and antibody, showed significantly different results between the two study groups, with the majority of patients with severe ADAMTS13 deficiency having reduced ADAMTS13 antigen and positive antibodies, whereas the majority of patients without severe ADAMTS13 deficiency having normal ADAMTS13 antigen and no antibodies. However, ADAMTS13 antigen was usually only moderately reduced in patients with severe ADAMTS13 activity deficiency and anti-ADAMTS13 antibodies were detected (although at a low titer) in one-fourth of patients with ADAMTS13 ≥ 10%, pointing out that the measurement of ADAMTS13 activity is the only useful test for TMA/TTP differential diagnosis.

As expected, patients with severe ADAMTS13 deficiency underwent a higher number of PEX procedures than those without severe deficiency. PEX was often discontinued when the ADAMTS13 activity test done by the central laboratory was negative. Thus, an early assessement of ADAMTS13 activity is warranted not only to choose the most effective treatment, but also to avoid unnecessary PEX, with the related side effects and costs. Kim et al. estimated the economic impact of the delay in obtaining pre-treatment ADAMTS13 values in patients admitted with a clinical presentation of TMA and showed that the availability of a rapid turnaround time for pre-PEX ADAMTS13 measurement is cost-effective, the incremental cost to the healthcare system being about $4155–5123 for each day of delay [27].

Pertaining to the outcomes of acute TMA events, our results on exacerbations and relapses demonstrate that ADAMTS13 activity testing identifies patients with different disease course. Those without severe deficiency showed a worse survival rate, which might be explained by the higher prevalence of concomitant cancer and older age. Conversely, exacerbations (39% of cases) and relapses within 6 months (17% of cases) occurred only in patients with severe deficiency.

The average annual incidence of acute TTP events estimated by us (1.2 cases per million people) is somewhat lower than previously reported: 1.5 in France [28], 2.1 in Germany [3], 3.10 [1, 16] in the USA, 6 in the UK [4]. Barring explanations such as differences across populations (e.g., prevalence of African-Americans) and mortality bias (patients who had died prior to being enrolled in the study), our incidence is most likely underestimated because only one third of all the transfusion centers located in Lombardy, representative of 50% of overall therapeutic plasma-exchange procedures, did participate to the study. Taking this into account, the annual incidence of acute TTP events increased up to 3.5 events per million people, which is still in the same order of magnitude as previously reported estimated of TTP incidence. Although likely underestimated, this is the first estimate of TTP incidence available in Italy and confirms the ultra-rare nature of this disease.

Our study demonstrates the feasibility of implementing a Hub and Spoke model of centralized, rapid testing of ADAMTS13 for the confirmation of TTP diagnosis in a vast Region of Northern Italy. This strategy may have several advantages and disadvantages. Advantages includes (i) the standardization and high quality of ADAMTS13 testing, especially when it is provided by laboratories with long-standing experience and highly specialized personnel; (ii) the networking of treating clinicians, which eases contacts with expert hematologists in the field (even more important in the era of new targeted therapies as caplacizumab); (iii) the optimization of resources both in terms of consumables and personnel. Conversely, disadvantages of this strategy includes (i) the logistic, administrative difficulties inherent in implementing such hospitals network, which, however, needs to be geographically limited to efficiently respond to the emergency needs of acute TTP patients; (ii) storage and transport issues, which require dry ice if samples cannot be received soon after collection; (ii) a significant burden on laboratory personnel, especially in case of out of hours tests.

This study has limitations. The main one is the small sample size, particularly for the non-TTP TMA group of patients, which led to inaccurate estimates of the analyzed enpoints with wide confidence intervals. Another limitation may be the referral pattern. Patients were treated in different transfusion centers and thus differences in clinical practice may have influenced disease management. Despite these limitations, this study suggests a feasible model of practical clinical approach to TMAs that was implemented in a large Italian Region and underlines the importance of ADAMTS13 activity testing for the optimal management of TMA patients.

## Conclusions

This multi-center study establishes the clinical need of ADAMTS13 activity testing in TMA patients requiring PEX therapy. Severe deficiency of the VWF cleaving protease allowed to confirm the diagnosis of TTP and identify two groups of patients with different disease courses. Thus, ADAMTS13 activity testing is strongly recommended for an accurate differential diagnosis and therapeutic approach of TMAs.

## Supplementary Information

**Additional file 1.** **Table S1.** Number of enrolled patients for each enrolling center; **Table S2.** Estimated incidence of TTP in the Region of Lombardy.

## Data Availability

The datasets generated during and/or analysed during the current study are available from the corresponding author on reasonable request.

## Abbreviations

ADAMTS13: A disintegrin and metalloproteinase with a thrombospondin type 1 motif member 13
BMI: Body mass index
CI: Confidence interval
eGFR: Estimated glomerular filtration rate
IQR: Interquartile range
LDH: Lactate dehydrogenase
PEX: Plasma-exchange
TMA: Thrombotic microangiopathy
TTP: Thrombotic thrombocytopenic purpura
VWF: Von Willebrand factor
