# Supplementary material for: Lombardy diagnostic and therapeutic network of thrombotic microangiopathy
Source: Orphanet J Rare Dis. 2022 Jun 23;17:246. doi: 10.1186/s13023-022-02400-y (PMC9229100; doi:10.1186/s13023-022-02400-y)
Supplement: Supplementary file 1 — Additional file 1. Table S1. Number of enrolled patients for each enrolling center; Table S2. Estimated incidence of TTP in the Region of Lombardy. [file 13023_2022_2400_MOESM1_ESM.docx]

**Supplementary Material**

**Lombardy diagnostic and therapeutic network of thrombotic microangiopathy**

Mancini I^1^, Agosti P^1^, Boscarino M ^1^, Ferrari B^2^, Artoni A^2^, Palla R^1^, Spreafico M^3^, Crovetti G^4^, Volpato E^5^, Rossini S^5^, Novelli C^6^, Gattillo S^7^, Barcella L^8^, Salmoiraghi M^9^, Falanga A^8,10^, Peyvandi F^1,2^, Lombardy AREU TMA Network.

**Table S1. Number of enrolled patients for each enrolling center.**

| **Center** | **Referring staff members** | **Patients (n)** |
| --- | --- | --- |
| IRCCS Ca' Granda Maggiore Hospital Foundation, Milan (MI) | Flora Peyvandi, Andrea Artoni, Barbara Ferrari, Roberta Palla, Ilaria Mancini, Pasquale Agosti | 11^a^ |
| Alessandro Manzoni Hospital, Lecco (LC) | Marta Spreafico | 6 |
| ASST Valle Olona, Busto Arsizio Hospital, Busto Arsizio (VA) | Giovanni Crovetti | 5 |
| ASST Grande Ospedale Metropolitano Niguarda, Milan (MI) | Elisabetta Volpato, Silvano Rossini | 5 |
| Papa Giovanni XXIII Hospital, Bergamo (BG) | Anna Falanga, Laura Russo, Luca Barcella | 5 |
| IRCCS San Raffaele Hospital, Milan (MI) | Salvatore Gattillo | 3 |
| Legnano Hospital, ASST Ovest Milanese, Legnano (MI) | Chiara Novelli | 3 |
| Carlo Poma Hospital, Mantova (MN) | Enrico Capuzzo | 2 |
| Maggiore Hospital Lodi, Lodi (LO) | Marco D'Agostino | 2 |
| Istituti Ospitalieri, Cremona (CR) | Porcari Moreno, Inzoli Alessandro | 1 |
| Sant'Antonio Abate Hospital, Gallarate (VA) | Pagani Ambrogio | 1 |

^a^ A total number of 43 patients were enrolled, with one patient experiencing two acute events during the study period.

**Table S2. Estimated incidence of TTP in the Region of Lombardy.**

| Year | First acute TTP events | Person-years | Incidence | Incidence per million people |
| --- | --- | --- | --- | --- |
| 2014 | 8 | 10,002,615 | 8.00 x 10^-7^ | 0.80 [0.25-1.35] |
| 2015 | 7 | 10,008,349 | 6.99 x 10^-7^ | 0.70 [0.18-1.22] |
| 2016 | 7 | 10,019,166 | 6.99 x 10^-7^ | 0.70 [0.18-1.22] |
| Mean annual incidence | | | | **0.73 [0.43-1.04]** |
| Year | **Acute TTP events** | **Person-years** | **Incidence** | **Incidence per million people** |
| 2014 | 12 | 10,002,615 | 1.20 x 10^-6^ | 1.20 [0.52-1.88] |
| 2015 | 10 | 10,008,349 | 9.99 x 10^-7^ | 1.00 [0.38-1.61] |
| 2016 | 13 | 10,019,166 | 1.30 x 10^-6^ | 1.30 [0.59-2.00] |
| Mean annual incidence | | | | **1.17 [0.78-1.55]** |

Person-years have been calculated multiplying the number of residents in the Italian Region of Lombardy in the years 2014-2016 according to the data of the Italian National Institute of Statistics by the observation time (1 year).
